# Colocalisation of lanthipeptide production with genetic exchange and defence systems across prokaryote genomes

**DOI:** 10.1186/s12864-025-12219-z

**Published:** 2025-12-18

**Authors:** David Hourigan, Colin Hill, R. Paul Ross

**Affiliations:** 1 APC Microbiome Ireland, Biosciences Institute, Biosciences Research Institute, College Rd, University College, Cork, Ireland; 2 School of Microbiology, University College Cork, College Rd, University College, Cork, Ireland; 3 Teagasc Food Research Centre, Moorepark West, Fermoy, Co. Cork, Moorepark, Ireland

**Keywords:** Bacteriocin, Lanthipeptide, Bacteriophage, Defence-system, Competence

## Abstract

**Background:**

Bacteriocin production is a widespread trait among bacteria and has been shown to have a role in bacterial competition in complex communities. Lanthipeptides are a class of modified bacteriocins that can have both antibacterial and signalling activities and rely on a number of genes encoding production, modification, regulation and immunity. This study aimed to investigate whether class II lanthipeptide gene clusters co-locate with other encoded apparently unrelated functions.

**Results:**

A total of 1,412 verified lanthipeptide biosynthetic gene clusters (BGCs) were analysed for their co-localisation with other functions over a 40 kb span. We found that genes involved in phage defence were among the most commonly located close to the bacteriocin BGCs. This phenomenon was found across species, such as *Paenibacillus larvae* and *Corynebacterium matruchotii* ATCC 33806, that have restriction modification (RM) systems. Anti-phage-defence proteins were also found in 1.2% of sampled regions and these include the anti-restriction protein ArdA. Genes related to bacterial competence were also discovered close to bacteriocin genes in genera such as *Bacillus*,* Enterococcus and Streptococcus*.

**Conclusion:**

This over-representation of genes encoding DNA defence systems and systems associated with the uptake of exogenous DNA near class II lanthipeptide gene clusters suggests an evolutionary rationale in which bacteriocin-mediated killing/lysis is linked to DNA uptake and horizontal gene transfer. The presence of anti-CRISPR proteins and RM-systems also suggests convergence of genetic systems that perpetuate their own survival through mutually-beneficial genomic co-localisation. This, coupled with recent evidence showing co-transcription of ribosomally-synthesised peptides and phage defence systems, suggests that the production of antimicrobial peptides forms part of a broader system where bacterial antagonism and competition is linked to horizontal gene transfer and competence as observed in streptococci.

**Supplementary Information:**

The online version contains supplementary material available at 10.1186/s12864-025-12219-z.

## Background

Bacterial genomes evolve through genomic rearrangements, mutations and horizontal gene transfer (HGT), with the latter contributing significantly to the diversity and adaptation of species. Genomic islands (GIs) represent regions of DNA that can harbour multiple operons, that can or have previously undergone HGT. These play crucial roles in the diversification of species through expansion of the accessory genome [1]. Multiple studies have characterised “defence islands” that are GIs harbouring multiple bacteriophage (phage) defence systems [2–5]. A recent study has uncovered a repertoire of nine novel anti-phage defence systems that are frequently clustered on defence islands, some of which were found to have undergone HGT [5]. The linking of these “gain events” through co-occurrence can uncover systems linked through co-selection where the gain of two simultaneous functions provides an additive and sometimes synergistic benefit to the host [6]. Gaining multiple systems co-localising on mobile genetic elements (MGEs) can be seen as an evolutionarily advantageous method of gene dispersal, in particular for selfish genetic elements. This rationale can be exploited to identify previously unknown systems in a “guilt by association manner” as seen with phage defence islands [4, 5]. GIs are not limited to phage defence and include pathogenicity islands, symbiosis islands, metabolic islands, resistance islands and fitness islands [1].

Antimicrobial peptide production is widespread across the bacterial kingdom and has multiple ecological roles including competition and signalling [7]. These peptides have tremendous diversity in terms of chemical structures and mechanisms of action. Bacteriocins are a group of ribosomally-synthesised peptides that are capable of both killing and communicating with other bacteria.Their production can be considered an offensive trait [7–9]. These peptides are broadly categorised into class I modified and class II unmodified bacteriocins. Indeed, class I bacteriocins are one of the few examples where extensive post-translational modifications occur in prokaryotes. Bacteriocin production has also been linked to traits such as sugar utilisation and competence [10–15]. An example of this can be seen among streptococci with the genetic linking of competence and bacteriocin production that promotes competition through co-ordinated expression [10, 12, 13]. Other genetically-linked traits include nisin production and the ability to utilise sucrose that are both encoded on the same conjugative transposon, but it remains unclear if this provides the host with an ecological advantage [15]. The nisin BGC co-localises on a conjugative transposon with a phage defence system and conjugation of the full element can confer all three abovementioned traits [15–17]. Similarly, phage-mediated horizontal gene transfer of the virulence-associated GI νSaβ included a bacteriocin gene cluster along with other niche adaptation virulence factors such as staphylococcal superantigens, leukotoxins and proteases [18].

Little is known of other traits that are associated with bacteriocin production on GIs or if the phenomenon is widespread across the bacterial kingdom. We set out to find as-yet-unknown traits that co-localise with the genes encoding the machinery responsible for the production of bacteriocins. These putative regions may have undergone coordinated HGT events. We examined a 20 kb genomic neighbourhood (both upstream and downstream) anchored at the *lanM* gene, which is responsible for the dehydration and cyclisation steps of core peptide maturation. These post-translational modifications introduce lanthionine ring formations which are essential for antimicrobial activity [19]. The length chosen is consistent with the size of elements capable of HGT either through transducing phage particles, integrative and conjugative elements (ICE), or plasmids [1, 5]. This window size is also in agreement with the window used to discover novel anti-phage defence systems [5]. However, in this study, we opted to examine a 20 kb neighbourhood either side of the *lanM* gene rather than analysing consecutive genes, as BGCs often contain multiple small open reading frames within a compact genomic region [20–22]. This region also encompasses the BGC along with an adjacent non-BGC neighbourhood of approximately the same size or greater.

**Fig. 1:**
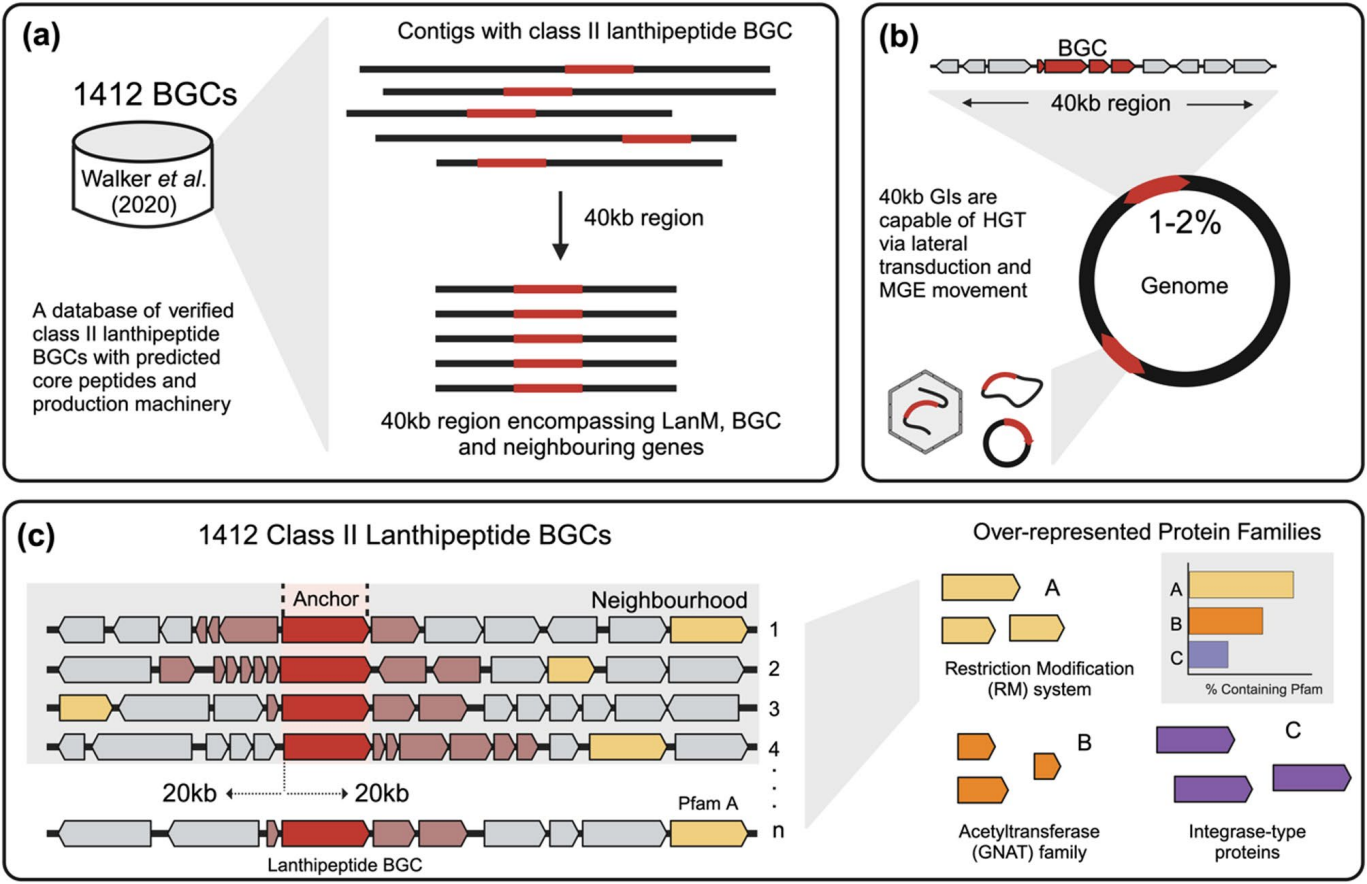
Overview of the search method. **a** LanM proteins were identified using a HMM model. Only previously detected BGCs were brought forward by cross-referencing data with Walker *et al*. [23] to reduce the number of false positives in the dataset. The presence of Lanthionine synthetase C-like protein domains (PF05147, encoded within the LanM gene) that exist in other proteins or other classes of BGCs can skew the dataset with false positive lanthipeptide BGCs. Regions were reduced to 40 kb in total length encompassing the *lanM* gene, the BGC and the surrounding gene neighbourhood. **b** Taking an average genome size to be between 4–5 million bp, the window search size is approximately 1% of the genome. This is slightly higher for genera with smaller genome sizes such as *Enterococcus* and *Lactococcus*. This region size and start location was chosen as it includes the BGC and regional DNA and is representative of a size of DNA that can move via transducing phage and MGE. This results in approximately 30 kb non-BGC DNA taking an average BGC to span 10kb. **(c)** Within the 40 kb regions, where n is the total number of regions, Pfams co-occurring in the genomic neighbourhood of class II lanthipeptide operons were identified. Shown is a visual representation of the over-representation of restriction-modification systems near class II lanthipeptide BGCs. Red represents BGC-associated genes with the *lanM* gene in darker red. Yellow represents genes encoding restriction-modification proteins and grey represent non-BGC genes without significant over-representation

We aimed to identify genes co-localised with bacteriocin BGCs by taking the class II lanthipeptide BGCs as a test case (Fig. 1). This type of BGC was chosen based on the recent discovery of over a thousand BGCs of this type and the presence of a single class-defining enzyme “LanM” [23].

## Results

### Construction of class II lanthipeptide BGCs protein family dataset

**Fig. 2:**
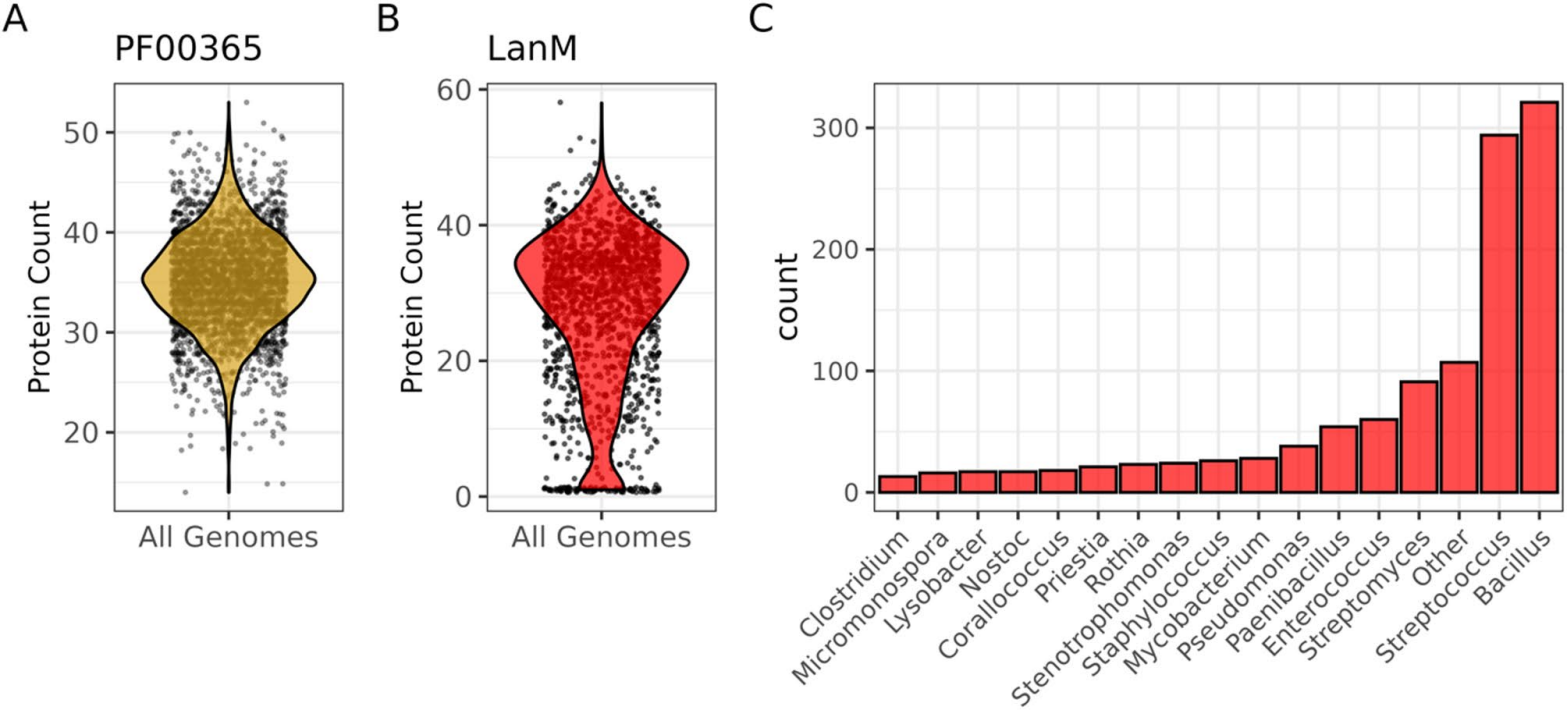
Organisation of the datasets (**a**) The control (gold) dataset consists of 3,148 contigs shortened to a window of 40 kb similar to the test dataset. This region is anchored to a gene encoding phosphofructokinase (PF00365) (**b**) The lanthipeptide test group (red) consist of 1,412 contigs encoding on average 37 CDS within 40 kb. The window region was set to a maximum of 40 kb and that window sizes exist between 2–40 kb. Both control (gold) and test (red) datasets have approximately the same mean coding density of 37 CDS per 40 kb window. **c** *Bacillus*, *Streptococcus*, *Streptomyces* and *Enterococcus* make up the top 4 genera with predicted class II lanthipeptide BGCs

A total of 14,494 lanthionine synthetase C-like protein hits were identified in the nr database [24]. To reduce the presence of false positives in the dataset, these were cross-referenced with the Walker *et al*. 2020 study resulting in 1,412 contigs with putative lanthipeptide BGCs. These BGCs were classified based on the presence of genes encoding a putative core peptide, transport machinery and a LanM protein [23]. Genes encoding phosphofructokinase (PFK, PF00365) were used as a completely random but ubiquitous Pfam class to create a control dataset for validation. Both class II lanthipeptide (test) and PFK (control) datasets have similar coding densities within the compared regions of approx. 37 coding sequences (CDS) per 40 kb window (Fig. 2a). Predicted BGCs were found to span a total of 189 different genera based on NCBIs taxonomy classification. The top five genera with genes encoding LanM-type BGCs were *Bacillus*, *Streptococcus*, *Streptomyces*, *Enterococcus* and *Paenibacillus*, all of which are genera with confirmed bioactive class II lanthipeptide BGCs (Fig. 2c). A total of 1,412 unique regions were analysed in total encompassing 4,343 distinct Pfam domains.

### Non-random clustering of protein families near class II lanthipeptide operons suggests evidence of horizontal gene transfer

**Fig. 3:**
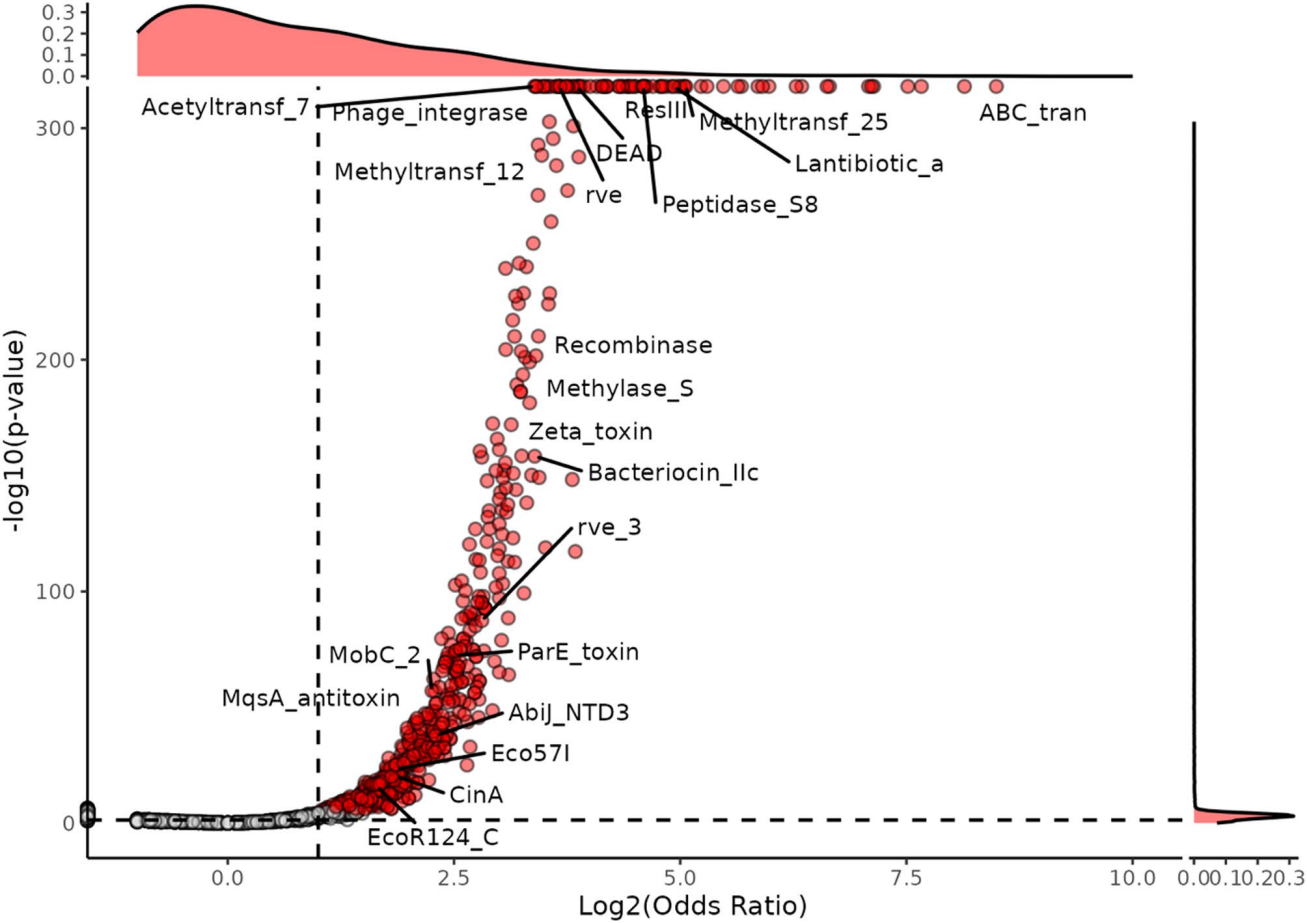
Over-represented Pfams near lanthipeptide BGCs. A scatter plot of -log10 (*p*-value) vs. the log2(odds ratio) for Pfam counts compared to a random distribution. Highlighted in red are Pfams which are statistically enriched compared to chance (Bonferroni adjusted *p*-value < 0.05 and Odds Ratio > 2). Dashed lines mark the significance threshold (*p*-value = 0.05) and an odds ratio of 2. The density plots on the x-axis and y-axis highlight the majority of Pfams don’t hold significance. Over-represented Pfams are commonly found encoded on proteins in class II lanthipeptide BGCs such as ABC transporter (PF00005, ABC_tran), domain of unknown function (DUF4135/LanC-like; PF13575), peptidase C39 family (PF03412) and the lanthipeptide mersacidin (PF16934). An over-representation of Pfams associated with the movement of DNA was also observed such as recombinase, type I restriction modification DNA specificity domain (PF01420, Methylase_S), integrase (PF13683, rve_3), phage integrase (PF00589) and bacterial mobilisation protein MobC (PF05713)

To assess whether Pfams associated with lanthipeptide BGCs exhibit non-random clustering, we performed random sampling of Pfams equivalent in size to the test dataset (Fig. S1). We then compared the observed Pfam counts to the expected count under a randomly sampled distribution to determine the likelihood of observing those counts by chance (Fig. 3). Pfams with a significant enrichment (Bonferroni corrected *p*-value < 0.05) and an odds ratio greater than 2 (> 2 fold likelihood of observing the Pfam in the dataset than chance) were highlighted in red. This analysis identified significantly over-represented Pfams near lanthipeptide BGCs, suggesting potential Pfam-attributed functional association to neighbouring genes. Genes encoding a total of 561 Pfams were found to be statistically over-represented near class II lanthipeptide BGCs as determined by Fisher’s test compared to chance (Table S1). We also compared the occurrence of Pfams near a BGC compared to PFK as a secondary random-gene control and found that 269 Pfams were enriched in the lanthipeptide dataset compared to both control datasets (Table S1-2, Fig. S2-S4). An expected abundance of Pfams associated with gene regulation such as cro/C1-type helix-turn-helix HTH DNA-binding domain (PF13443) and helix-turn-helix domain (PF13560) were observed which likely play a role in the regulation of the BGC or operons in the BGCs genomic neighbourhood. Pfams also over-represented were ABC transporter transmembrane region (PF00664), peptidase C39 family (PF03412) and lantibiotic alpha (PF14867). A second observation was the over-representation of mobilome-associated Pfams such as the transposase DDE domain (PF13751), phage integrase, SAM-like domain (PF13102), recombinase (PF07508), bacterial mobilisation protein (MobC) (PF05713) and transposase IS66 family (PF03050). A third observation was the over-representation of Pfams associated with defence against exogenous DNA and HGT (Fig. 3). To investigate the relationship between region counts and Pfam abundance, we randomly subsampled 100 random BGCs and their surrounding 40 kb window and observed that as the number of regions sampled increases, the number of detected Pfams also rises, demonstrating a positive correlation between increasing sample size and increased detection of co-localised Pfams (Fig. S3). This was also the case for randomly chosen negative controls, which showed little to no correlation (*R* < 0.31). Strong positive correlations were observed for BGC machinery such as the peptidase C39 family (PF03412) which cleaves double-glycine motifs (*R* = 0.99). Positive correlations were observed for parE toxin of type II toxin-antitoxin system, parDE (PF05016) (*R* = 0.73, *p* < 2.2e-16), zeta toxin (PF06414) (*R* = 0.63, *p* = 1.2e-12), type I restriction modification DNA specificity domain (PF01420) (*R* = 0.78, *p* < 2.2e-16), phage integrase (PF00589, *p* < 2.2e-16) (*R* = 0.83), GNAT acetyltransferase (PF00583) (*R* = 0.84, *p* < 2.2e-16), recA bacterial DNA recombination protein (PF00154) (*R* = 0.75, *p* < 2.2e-16). A table of HGT-related Pfams that co-occur within BGCs and their frequencies is found in Table 1. As we observed Pfams commonly associated with MGE, we predicted the presence of plasmid and GI-localised BGCs. We then observed that 14.8% (209/1412) of BGCs were predicted to be encoded on plasmids and 10.8% (156/1412) were predicted to be localised within GIs (Table S3, Table S10). Specific cases were then chosen for further investigation based on ecological roles (defence, mobilome, competence) and statistical over-representation.

**Table 1:** Over-represented protein families associated with the movement of DNA and bacterial fitness

Class		Name	Pfam	%BGC	BGC (*n* = 1412)	%Control	Control (*n* = 3148)
BGC	Lanthionine synthetase C-like protein		PF05147	100	1412	< 0.1	1
Control	Phosphofructokinase		PF00365	0.6	9	100	3148
TA-system	ParE toxin of type II toxin-antitoxin system, parDE		PF05016	5.2	74	0.1	3
TA-system	Zeta toxin		PF06414	4.7	66	0.1	2
TA-system	RelB antitoxin		PF04221	4.6	65	0.1	3
TA-system	Antitoxin, MqsA		PF15731	3.0	43	0.1	3
RM-system	Type I restriction modification DNA specificity domain		PF01420	5.5	78	0.3	11
RM-system	Eco57I restriction-modification methylase		PF07669	3.6	51	0.8	24
RM-system	Type I restriction enzyme R protein N terminus (HSDR_N)		PF04313	3.5	48	0.3	9
Anti-phage helicase	SWI2/SNF2 ATPase		PF18766	4.9	69	0.5	15
Acetyltransferase	Acetyltransferase (GNAT) family		PF00583	14.0	198	5.3	169
Acetyltransferase	Acetyltransferase (GNAT) family		PF13508	11.0	159	2.3	71
HGT	Resolvase		PF00239	8.2	116	0.4	14
HGT	Phage integrase family		PF00589	9.1	129	3.5	110
HGT	Integrase core domain		PF00665	7.0	99	1.1	36
HGT	Recombinase		PF07508	3.0	43	0.3	8
HGT	Transposase DDE domain		PF01609	3.6	52	0.5	16
HGT	Bacteriophage CI repressor helix-turn-helix domain		PF13102	3.1	44	< 0.1	1
HGT	Phage antirepressor protein KilAC domain		PF03374	1.3	18	< 0.1	1
Competence	Competence-damaged protein		PF02464	3.2	46	0.4	12
Recombination	recA bacterial DNA recombination protein		PF00154	3.4	48	1.4	43
Quorum sensing	RNPP family C-terminal domain		PF18768	3.3	47	< 0.1	1
Metabolism	Beta-L-arabinofuranosidase, GH127		PF07944	6.6	93	0.1	3
Metabolism	Glycosyl hydrolase family 1		PF00232	5.7	81	0.5	15
Metabolism	Phosphomethylpyrimidine kinase		PF08543	7.1	100	0.9	28
Metabolism	PTS system, Lactose/Cellobiose specific IIB subunit		PF02302	4.2	59	0.2	7
Metabolism/stress response	Glucose inhibited division protein A		PF01134	4.5	63	0.8	25
BGC-(NRPS)	Condensation domain		PF00668	3.7	95	0.3	11

### Non-random clustering of protein families indicates the co-localisation of fitness traits and addictive modules with BGCs

To determine whether specific protein families associated with BGCs are linked to particular functions, we cross-referenced co-localised Pfams with Doron *et al*. (2018) (phage defence dataset), the mobile orthologous groups (mobileOG), Distilled and Refined Annotation of Metabolism (DRAM) and Gene Ontology (GO) databases [25–27]. It is important to recognize the limitations of GO function mapping, as annotations can be either overly broad or excessively specific, sometimes lacking meaningful biological insight. Therefore, this was used to further guide downstream analysis rather than inferring statistically over-represented functions. In summary, 101, 45 and 35 Pfams were found over-represented in this dataset and found in the mobileOG database, DRAM database and Doron *et al*. (2018), respectively (Fig. S5). Genes encoding multiple restriction endonuclease domain-containing Pfams (PF04313 and PF04471) were also over-represented (Table. S1, S2). Notably, genes encoding other Pfams associated with phage defence and toxin-antitoxin (TA)-systems including the parE toxin of a type II TA-system (ParDE, PF05016) were over-represented [28]. However, only two of these were predicted to be localised to plasmids (NZ_ALKW01000013.1 and NZ_KB932388.1). Zeta toxins are part of the diverse type II TA-system, and a recent study has elucidated their role in interrupting phage infection by inhibiting protein synthesis following chaperone-mediated recognition of viral structures [29]. Genes encoding a type I RM DNA specificity domain (PF01420, Methylase_S) co-localises with 5.6% of BGCs (78/1412) (Fig. 3; Table 1). This is the specificity subunit of type I RM systems and was found in 0.35% of regions in the control dataset and 12 times in the shuffled-random dataset (10 to 15-fold difference) (Fig. 3, Table S1, Table S2). This domain was found on 37 non-redundant proteins across 8 distinct species including *Trueperella pyogenes* (NZ_CP012649.1), *Streptococcus pyogenes* ATCC 10,782 (NZ_GL397225.1), *Streptococcus pneumoniae* GA44128 (NZ_AIKV01000003.1), *Arthrobacter* sp. ok362 (NZ_FNGC01000009.1), *Paenibacillus larvae* subsp. *larvae* (NZ_CP019655.1) and *Corynebacterium matruchotii* ATCC 33,806 (NZ_EQ973330.1). Over 5% of BGC regions encoded a type I restriction modification-type system associated Pfam (78/1412) (Fig. 3, Table S1, Table S2). Genes encoding an N-6 DNA methylase protein (PF02384) were found in *Streptococcus*, *Paenibacillus*, *Arthrobacter*, *Corynebacterium* and *Trueperella*. In total 87.0% (1229/1412) of regions contain genes encoding a Pfam previously shown to be part of a phage defence system using the supplementary data from Doron *et al*. (2018). This is inflated by the number of AAA domains (PF13304, *n* = 1536). Removing AAA-domain-like Pfams results in 40% (561/1412) of regions having a defence mechanism-associated Pfam. To account for spurious domain assignment and multiple domains residing on a single protein, regions with five or more Pfams not including AAA-related Pfams associated with defence were brought forward. In total, 88 regions had five or more genes encoding defence-associated Pfams spanning 36 genera. Similar defence-associated Pfams and systems are also dispersed across genera such as genes encoding eco571 RM methylase (3.6% of BGCs) which can be seen in *Streptococcus*, *Clostridium*, *Enterococcus*, *Leuconostoc* and *Flavobacterium* (Fig. 3, Table S5). A previous study identifying anti-phage defence systems highlighted the presence of genes encoding multiple Lon protease; phospholipase D-like domain at C-terminus of MIT (PF16565), BrxL -Lon-like protease (COG4930) and putative ATP-dependent Lon protease (PF13337) as part of the BREX anti-phage defence system [30]. The statistically over-represented Pfams archaeal LonB, AAA + ATPase LID domain (PF20436) and Lon-like LonC helical domain (PF20437) were also found over-represented often encoded with a protein encoding a methyltransferase domain (PF13847, Methyltransf_31) resembling the BREX anti-phage system although composed of different Pfams. Over 14% of BGCs (198/1412 BGC) were found to be localised with a gene encoding a GCN5-related N-acetyltransferase (GNAT) family Pfam compared to 5.4% of the PFK dataset (Fig. S6). Acetyltransferases also shared no significant percentage identity to PaeN (AFS60110.1), an acetyltransferase encoded in the operon of the N-terminally acetylated class I lantibiotic paenibacillin. As acetyltransferases are known to confer antibiotic resistance to aminoglycosides, each protein was searched for homology to known AMR genes in the CARD database. However, only a single and non-GNAT acetyltransferase protein had greater than 70% identity with the AAC(6’)-Ie-APH(2’’)-Ia bifunctional protein (WP_027622104.1, 99.4% ID, E-value 2.32e-134). This protein confers aminoglycoside resistance and was found localised near a single BGC found in *S. suis* (NZ_CDUL01000018.1). Due to the vast functional diversity of GNAT acetyltransferases it is unlikely that these proteins function as antibiotic resistance genes and instead function as toxin modules. Acetyltransferases have been found co-localised with TA-systems and other toxin-like proteins in “toxin islands” like the clustering of anti-phage systems in “defence islands” [31]. Toxin-like function was predicted for 6.6% (13/197) GNAT acetyltransferases as determined by homology to proteins in the toxinome database (E-value < 1e-05). Acetyltransferases have also been shown to confer anti-CRISPR activity (AcrVA5, WP_046699157.1) [32, 33]. However, only two sequences shared low homology with known anti-CRISPR acetyltransferases AcrVA5 (WP_046699157.1) and GNAT acetyltransferase (WP_095838468.1) (E-value < 1e-04 and >29% identity) (Table S8, Fig. S6). Two significant matches were found to the *Phikmvvirus* phage encoded GNAT (WP_068613667.1 and WP_213620502.1, E-value < 1e-10) and both matches in this dataset were confirmed to be of phage origin due to phage structural proteins present in the region and shared identity to predicted toxins [33].

### Co-localisation of DNA uptake systems with class II lanthipeptide BGCs

**Fig. 4:**
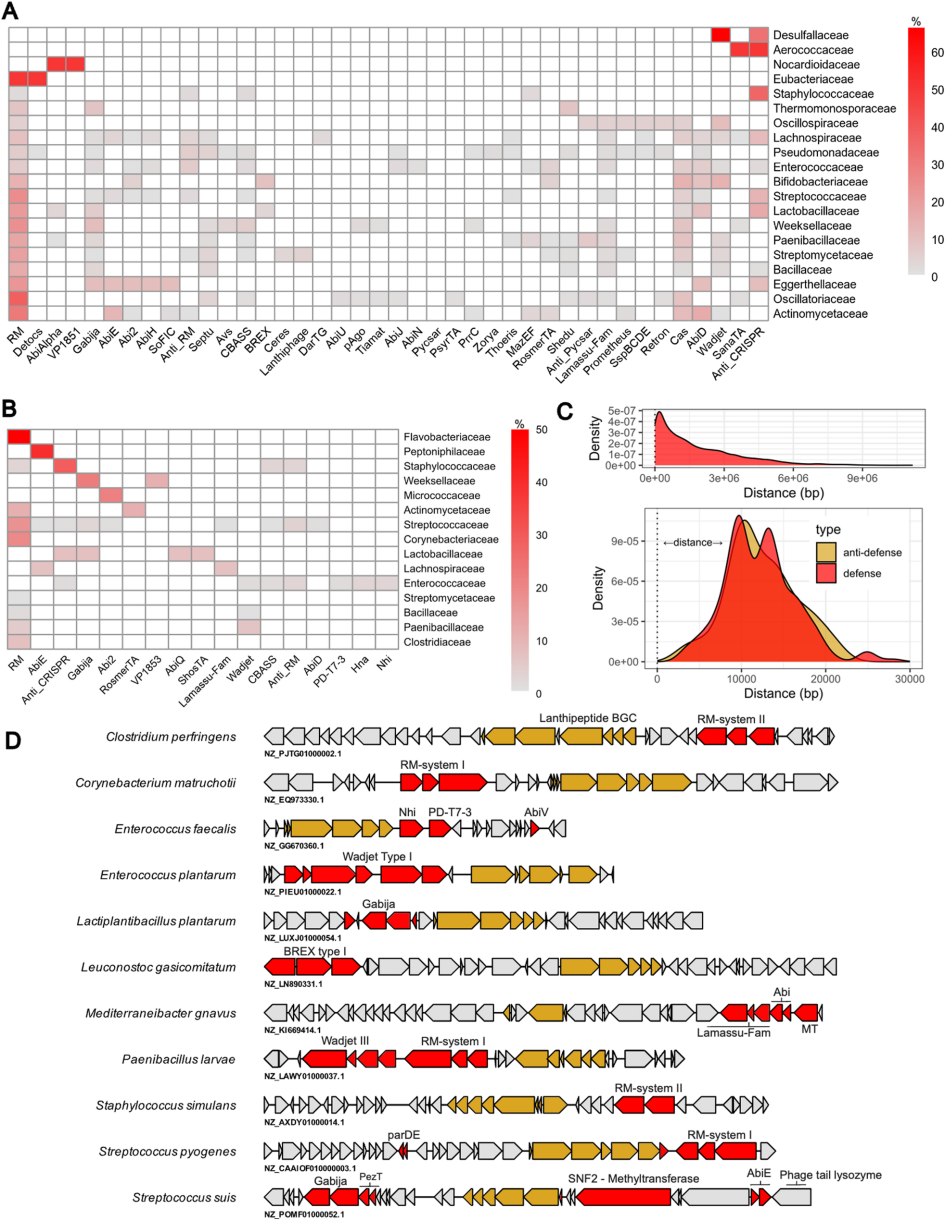
RM-systems are localised near class II lanthipeptide BGCs. **a** A heatmap showing the co-localisation of defence-systems and anti-defence systems on contigs encoding BGCs. Shown are the top 20 families of bacteria with lanthipeptide BGCs and defence systems predicted by DefenseFinder. The percentage of each family that contains each defence system on the contig encoding a class II lanthipeptide BGC is shown. **b** Shown are systems found localised within 20 kb of a gene encoding a LanM protein and the percentage of each family that encode each defence system. **c** Shown is the distance of defence and anti-defence systems from the LanM protein (x = 0 bp) across all contigs (top) and within each 40 kb window (bottom). Distance was defined as the midpoint of lanM and midpoint of each defence-associated gene. **d** Genes in red encode proteins from systems predicted by DefenseFinder that are over-represented near lanthipeptide operons. Genes in gold encode LanM, core peptides, LanP and regulatory elements LanR and LanK. Abi refers to abortive infection proteins and MT refers to methyltransferase proteins

As we observed an over-representation of Pfams associated with the HGT we then predicted the presence of phage defence systems and anti-CRISPR proteins on each contig (Fig. 4a). In total 40.1% (577/1412) and 12.4% (175/1412) of contigs containing a BGC encoded a defence system and anti-defence system, respectively (Table S6, S9). This was then curated to look within the same 40 kb region including the BGC, and RM-systems, anti-CRISPR proteins and anti-RM proteins were found (Fig. 4b). In total, genes encoding 17 anti-defence proteins and 136 defence systems were found localised within approximately 10 kb of *lanM* genes (Fig. 4c). We then took a random 30 consecutive coding sequences (CDS) from 200 random bacterial genomes, equivalent to the region of our test dataset that doesn’t encode the BGC and found 6 encoded an anti-phage defence system (an average of 37 CDS per region minus ~ 7 CDS for the BGC). This corresponds to an approximately fivefold greater likelihood of observing an anti-phage defence system in proximity to a lanthipeptide BGC compared to a randomly selected genomic region with similar non-BGC coding density (odds ratio = 4.90, 95% CI: 2.17–13.72; *p* = 3.7 × 10⁻⁶) (Fig. S7). To further illustrate the diversity of anti-phage defence systems found near lanthipeptide BGCs, we examined specific examples from our dataset (Fig. 4d). *Streptococcus pyogenes* (NZ_CAAIOF010000003.1) encodes a type I RM and *Enterococcus plantarum* TRW2 (NZ_PIEU01000022.1) encodes the Wadjet anti-phage defence system composed of the DUF220 family protein, DUF4194 and SbcC domain-containing protein (Fig. 4d). *S. pyogenes* (NZ_CAAJDZ010000002.1) was particularly rich in Pfams associated with phage defence with eleven such genes within the 40 kb region. The region encodes a complete type II RM system downstream from the lanthipeptide operon, an Eco47II family restriction endonuclease and methyltransferase upstream and the region also encodes a relE/parE type TA-system. Genes encoding RM systems with a DEAD/DEAH box helicase family protein, restriction endonuclease subunit S and class I SAM-dependent DNA methyltransferase were found in *Paenibacillus larvae* (NZ_CP019655.1) and *Corynebacterium matruchotii* ATCC 33,806 (NZ_EQ973330.1). (Fig. 4d). This system resembles the Defence Island System Associated with Restriction-Modification (DISARM) phage defence system. This 40 kb window also contains two N-6 DNA methylase-type proteins between the Wadjet system and the lanthipeptide BGC suggesting co-localisation of multiple systems. *Leuconostoc gelidum* subsp. *gasicomitatum* KG16-1 encodes the BREX anti-phage defence system (Fig. 4d).

Interestingly no genes encoding Clustered Regularly Interspaced Short Palindromic Repeats (CRISPR) Cas defence systems were found to be over-represented even though these systems were present in the BGC dataset.

**Fig. 5:**
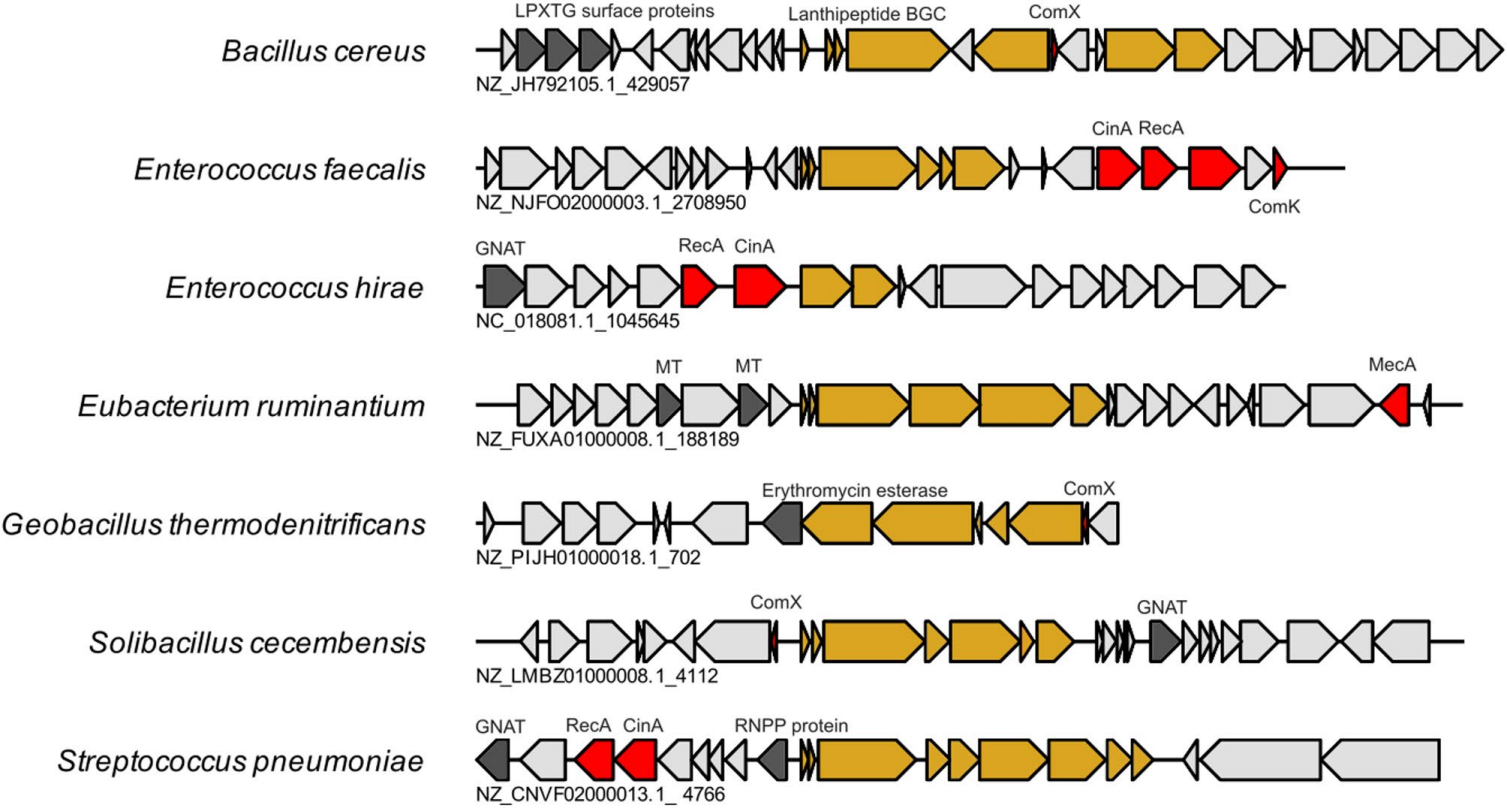
Gene encoding competence-associated Pfams are localised near class II lanthipeptide BGCs

Multiple genera have competence-associated genes localised near their respective lanthipeptide operon. These include *Bacillus cereus*, *Enterococcus hirae Streptococcus pneumoniae*,* Bacillus thuringiensis*,* Solibacillus cecembensis*,* and Geobacillus thermodenitrificans* (Fig. 5). The competence gene *comX* was solely found in Bacillaceae. The ComX pheromone induces the cellular transition to a competent state, thereby influencing DNA uptake and increasing the likelihood of integrating exogenous DNA [34]. Other competence-associated Pfams were also identified near lanthipeptide BGCs. These included cinA (PF02464, competence-damaged protein) and domain CinA_KH (PF18146, damage-inducible protein CinA KH domain), which are often located adjacent to *recA* (PF00154) (Fig. 5). RecA catalyses the ATP-dependent DNA strand-exchange reaction that is central to homologous recombination and double-strand break repair (Fig. 5). In addition, MecA (PF05389), a negative regulator of genetic competence, was detected in the dataset but was not statistically over-represented near BGCs (Fig. 5).

Genes encoding the Pfam Bacteriocin class II with double-glycine leader peptides (PF10439) were also found and these were mirrored in the GO function mapping under “GO: defence response to gram-positive bacterium” but after manual inspection the majority of these mapped to putative lanthipeptides (17/28 unique proteins are putative class II lanthipeptides).

Genes encoding the *Bacillus* competence pheromone ComX (PF05952) were solely found in the order Bacillales. Whereas genes encoding competence-inducible protein A (CinA) and recombinase (RecA) proteins were found among the naturally-competent genera *Enterococci* and *Streptococci*. *S. pneumoniae* (NZ_CNVF02000013.1) encodes a class II lanthipeptide BGC situated within 5 kb of putative *cin*-like operon. This operon includes a gene encoding an LCP family protein that might play a role in restructuring the cell wall during competence [35]. Downstream from the *recA* gene is a gene encoding an N-acteylmuramoyl-L-alanine amidase (LytA), likely acting as an autolytic component involved in competence as seen in other streptococci [36].

## Discussion

This study concludes that genes encoding systems involved in DNA defence and DNA acquisition are often localised near class II lanthipeptide BGCs (Figs. 4 and 5). These systems include RM systems and competence systems. Pfams associated with competence included in the criteria for association were RecA bacterial DNA recombination protein (PF00154, *n* = 51) and the *Bacillus* competence pheromone ComX (PF05952, *n* = 22). DNA repair and maintenance proteins have recently been shown to co-occur with bacterial immune systems highlighting the inter-connected nature of protection against DNA and controlling acquisition of DNA [37]. A study identified that the toxin YafO, type II toxin-antitoxin system (PF13957) (over-represented in this dataset) was also found to be frequently encoded in the leading region of plasmids along with anti-defence systems [38]. An interesting finding in this study is over-represented of Pfams that are described in anti-phage defence systems [5]. A recent investigation of the ocean microbiome identified a co-transcriptional association between RiPPs and neighbouring protein families (within 50 kb), including anti-phage defence systems and MGE-associated protein families, indicating that these elements are frequently co-expressed [39]. Gao *et al*. also highlighted cases of lanthipeptide, lassopeptide and Radical S-adenosyl-L-methionine (rSAM)-type BGCs co-localised with anti-phage defence systems supporting the co-localisation of defence systems and different RiPP BGCs families [39]. That study observed co-expression of BGCs and defence systems and suggest that RiPPs may play a role in phage interactions [39]. Abi-like proteins can also include metalloproteases (Type II CAAX prenyl endopeptidase Rce1-like) that are involved in Abi during phage defence and two previous studies reported the co-localisation of genes encoding Abi-like proteins near bacteriocin gene clusters and used it a driver sequence to find seven novel bacteriocin gene clusters [40, 41].

This begs a question – what is the ecological relevance of the co-localisation of genes encoding phage defence systems near those encoding antimicrobial peptide production and what selective pressure(s) might be involved? The localisation of anti-defence proteins most likely facilitate the movement of MGE/BGCs to novel hosts acting as counter-defence as seen in the leading strand of plasmids [38]. The first and most likely rationale for the localisation of defence systems is that the acquisition of a mobile genetic element (MGE) with both bacteriocin production and phage defence systems provides an un-linked but mutually-beneficial set of traits to the new host. We hypothesise that the benefits of each system could be temporal and independent or transcriptionally linked, as observed by Gao *et al*. [39]. The expression of the bacteriocin operon may help the recipient bacterium to compete, whereas at another moment in time, the expression of a phage defence system facilitates survival during phage infection. Both may be particularly important for a bacterium invading a new ecological niche along with the capacity to utilise new carbon sources (Fig. S8). Although our analysis focused on class II lanthipeptides, it is worth considering whether similar co-localisation patterns occur in other bacteriocin classes. This phenomenon is seen in the conjugal transfer of an ICE encoding the class I lanthipeptide nisin BGC which can also confer increased phage resistance [15–17]. Gao *et al*. reported that RiPPs such as lassopeptides, sactipeptides, and radical SAM-dependent peptides have also been observed in close genomic proximity to phage defence systems [39]. The Gamma-Mobile-Trio system is an MGE that is rich in both offensive (T6SS) and anti-phage defensive systems and highlights that co-localisation of genetic systems with offensive and defensive functions likely increase the benefit of maintaining the MGE [42]. These elements have diverse defensive cargo including the Gabija system, RM-systems and Lamassau-Fam-type systems, all of which were found localised near class II lanthipeptide BGCs in this study. A similar phenomenon termed ‘multi-conflict islands’ has been highlighted where similar genetic cargo co-localise within GIs within *Serratia* species [43]. Bacteriocin BGCs also provide selective pressure to retain MGEs and their gene content since any cell losing the BGC will be killed by the antimicrobial produced by a cell that retains it. This phenomenon is observed with plasmid-encoded microcins produced by *Escherichia coli* and has been engineered into gram-positive plasmids to increase retention within *Lactococcus lactis* hosts with the expression of the bacteriocin lactococcin 972 [44, 45]. Both RM-systems, toxin-antitoxin systems, and bacteriocin BGCs can be described as addictive or selfish genetic elements rendering the host dependant on gene presence to prevent post-segregationally killing [46, 47]. Interestingly, these were among the most abundantly co-localised systems suggesting co-localisation of “addictive” genetic modules (Table S1). While further systematic analyses are needed, our findings suggest that bacteriocins of diverse classes may be recurrently embedded within ‘multi-conflict islands’, where offensive, defensive, and mobility functions are co-localised.

Another hypothesis is that selective pressure has driven these systems to co-localise as a result of additive selective pressure driven by ecological context. Bacteriocins are most effective against closely-related strains, allowing the producing strain or recipient host to eliminate competitors that share a similar ecological niche or engage in intra-species or intra-strain competition (fratricide) [10, 12, 13, 48, 49]. Jeopardising the viability of a close neighbour through competition could potentially induce prophages in that neighbour that could attack the bacteriocin-producing strain [50]. It is also possible that other species that are competing are actively going through chronic phage infection and coming into contact and killing them will leave the incoming bacteria susceptible to phage attack [51, 52]. Linking may also occur through a “guard down” anticipation mechanism, where simultaneously facing phage infection and bacterial competition could overwhelm a strain, leading to total population collapse due to the combined external pressures (Fig. S9). However, genetic linking of both systems would allow the bacterium to persist. Evidence supporting this ecological context has recently been described in the ocean microbiome [39]. A third hypothesis is that lanthipeptides are directly involved in phage defence. Evidence for this is highlighted by the discovery of “lanthiphages” which are LanBC-type class I lanthipeptide BGCs that have evolved to have anti-phage function found solely in the phylum Actinomycetota (Actinobacteria) and localised within defence islands [53]. Enveloped viruses such as RNA viruses have phosphatidylethanolamine (PE) lipids in their envelope that are derived from host cell membranes. The lanthipeptide duramycin reduces the efficacy of multiple enveloped viruses including West Nile, dengue and Ebola viruses by interacting with PE and phosphatidylserine (PS) receptors [54]. This suggests that class II lanthipeptides could have efficacy against enveloped bacteriophage. However, existing literature surrounding membrane-containing bacteriophage associated with gram-positive bacteria is limited to the family Tectiviridae, a family with two described phage targeting *Bacillus thuringiensis* [55–57]. *Chitinophaga pinensis* DSM 28,390 is a gram-negative producer of pinensin, a lanthipeptide with anti-fungal activity [58]. This, along with activity against enveloped viruses, suggest the range of functions attributed to lanthipeptide production may be greater than currently appreciated.

Interestingly, competence is another trait associated with the acquisition of foreign DNA. Competence-associated genes were also found co-localised near genes responsible for class II lanthipeptide production (Fig. 5). Competence in *Bacillus* sp. is regulated by a network of proteins involving ComQXPA, ComSK and MecA where the fate of the cell is decided using quorum sensing [34]. Ultimately higher concentrations of the quorum-sensing peptide ComX (PF05952) increases the probability of a transition to a competent state [34]. Genes encoding ComX proteins were found localised close to BGCs in 22 genomes (*n* = 0, PFK control dataset) including *B. cereus*, *B. thuringiensis*, *Bacillus toyoensis*, *Geobacillus thermodenitrificans*, *Paenibacillus* sp. and *Solibacillus cecembensis*. ComX is variable in amino acid sequence but usually contains a tryptophan residue in the C-terminal end of the peptide [59]. All ComX proteins identified have this conserved residue (Fig. S10). The location of *comX* near these operons suggests trans-acting coupling of competence with bacteriocin production. Quorum sensing responses are often linked to control of competence, biofilm formation and extracellular DNA release in *Bacillus* [59]. Extracellular DNA can result from autolysis/fratricide or gene transfer agents. It is likely that bacteriocin production acts as a fratricidal peptide in this phenomenon as seen in *Streptococcus* [10, 12, 60, 61]. This phenomenon has been observed in *Streptococcus salivarius* and unmodified class II bacteriocins where synchronised predation and competence is more widespread than previously anticipated [62].

Another genetic determinant associated with competence is the competence-damaged protein (CinA, PF02464). It is encoded by the first gene in the competence-inducible (*cin*) operon and is involved in transformation [61]. The CinA protein was found in 46 genomes including *Actinoalloteichus fjordicus*, *Corynebacterium* sp. HMSC05E07, *E. faecalis*, *E. hirae* and *Streptococcus pneumoniae* GA60190 (Fig. 5). This genetic determinant is also found close to *recA*, encoding a recombinase that can mediate homologous recombination and DNA repair, in *Enterococcus* and Streptococcus *species* (Fig. 5). In contrast, *comX* was observed in *Bacillus* genomes which highlights that competence-associated determinants, while genus-specific in their distribution, co-occur with lanthipeptide loci.

Novel DNA can provide a host with new functions [63]. Similarly, DNA found in the environment can act as templates for fixing damaged DNA and DNA can also be used as a source of essential nutrients such as carbon, nitrogen and phosphorous [63]. Therefore DNA is a desirable commodity in the microbial world and it makes sense to both protect the cell from DNA and also compete for it depending on the context. However, accessory genetic systems need to perpetuate their own-survival and genomic co-localisation is an advantageous mechanism to increase the likelihood of their co-inheritance and co-selection, thereby ensuring stable maintenance. This is termed genetic hitchhiking and suggests that apparently unrelated systems which co-localise can relay to ‘hidden’ functions that are ecologically linked [64]. This gene co-occurrence phenomenon warrants further investigation to decipher the ecological roles of lanthipeptides in microbially complex environments and the selective pressure these antimicrobial peptides hold on neighbouring genetic cargo.

## Conclusion

This study identified an abundance of MGE-associated genes, phage defence-associated genes, RM-systems, TA-systems and competence-associated genes near lanthipeptide BGCs suggesting that the relationship between bacteriocin production and acquisition or protection from exogenous DNA may be functionally or ecologically linked.

## Methods

### Search for LanM homologs and creation of dataset

The protein family lanthionine synthetase C-like protein (PF05147) was download in FASTA format, aligned with muscle v5.1 and compressed to create a HMM model using HMMER v3.3.1 [65]. The model was used to search the nr database for Lanthionine synthetase C-like proteins resulting in 14,494 hits. Rodeo v2.3.4 was used to retrieve the genomic context of these proteins with the settings “-j 1 -min 20 -ft ‘nucs’ -fn 25000 --evaluate_all”, resulting in returned contigs with a maximum length of 40,000 bp and small open reading frames (smORFs) called with a minimum length of 20 [66]. Rodeo annotated each CDS using Pfam v33.1 and the output was saved as a tab separated value (TSV) file. The nucleotide accessions of this search were filtered to include only hits discovered by Walker *et al*. [23] to work with a dataset of known class II lanthipeptide BGCs. Contigs were shortened to only include a 40 kb window. The taxonomy was appended to each data frame using the R package taxonomizR v0.10.5 which pulls taxonomic data from NCBI. This was then plotted using ggplot2 R package v3.5.1 to visualise the taxonomic distribution of BGCs.

### Creation of a control dataset (Phosphofructokinase, PFK)

The protein family PF00365 was downloaded and compressed into HMM models as previously mentioned. This was then searched into RefSeq database [24]. In order to account for biases in the database of known lanthipeptide BGCs only control contigs from the phyla with lanthipeptide BGCs were used and non-bacteriocin containing genomes. If an assembly was found in the test dataset (lanthipeptide positive) it was removed from the control dataset. If an assembly has a bacteriocin encoding gene found as a hit to the BAGEL4 database, it was omitted [67]. Bacteriocins were identified using the BAGEL4 database and BLASTp v2.11.0 + with an E-value of 1e-5 [68]. Pfam annotation was mapped to each protein coding sequence using Pfam v33.1 (downloaded on 04/03/22) and mmseqs2 v14.7e284 [69, 70]. All proteins were clustered using the mmseqs “cluster --cov-mode 1” and a redundancy filter of “--min-seq-id 0.95” was used with the ‘clusthash’ command. The representative sequences from each cluster were annotated using the Pfam database and the command mmseqs search with the parameters “-k 6 -s 7.5”. Members of the same cluster was subsequently annotated with the Pfam attributed from their representative sequence. 40 kb regions around each control dataset were then selected to mirror the test dataset.

### Determining co-occurring Pfams with class II lanthipeptides compared to chance

To evaluate whether Pfam domains associated with lanthipeptide biosynthetic gene clusters were enriched beyond what would be expected by chance, we performed a permutation-based statistical analysis. First, we identified Pfam domains in the lanthipeptide regions. We filtered this list to retain only Pfams beginning with “PF”. The total number of genes within these Pfam families associated with lanthipeptide clusters was calculated. To generate a null distribution, we randomly sampled Pfam identifiers with replacement from the Pfam pool using “stats” form R v4.4.2. We sampled from the pool of all Pfams observed, while maintaining the observed number of Pfams per region. This process generated an expected count for each Pfam under the null hypothesis of random association. Observed Pfam counts were compared to expected counts derived from the random sampling. This was repeated across 10,000 iterations to build an empirical null distribution for comparison. The empirical *P*-value was corrected by Benjamini-Hochberg (BH) method using “stats” v4.4.2 in R.

### Determining co-occurring Pfams with class II lanthipeptides compared to PFK

Fishers exact test was performed on the Pfam occurrence table to find statistically relevant counts of Pfams over-represented near BGCs. Fishers exact test was chosen due its applicability to uneven datasets and its application in Scoary, a tool which scores gene presence/absence in determining phenotype-genotype relationships for bacterial genome wide association studies [71]. A Bonferroni correction was applied to *P*-values to adjust for multiple comparisons and reduce the false-positive hit rate and only hits with a positive proportion difference and a *P*-value < 0.05 were kept. The proportion difference was calculated for both the control and test dataset and only a positive proportion difference was kept i.e. higher proportion of Pfams with class II lanthipeptides than the control implies directionality of statistical co-occurrence. A volcano-like plot was used to plot statistically significant Pfams with the -log10(Adjusted *P*-Value) plotted vs. log10(Proportion difference). This was plotted using ggplot2 v3.5.1. Pfams that occurred across multiple different species and higher level order were further investigated.

### Calculation of observed vs. expected counts

To determine co-localisation of Pfams within 40 kb of class II lanthipeptides a count table was created for both control and test datasets that consisted of the columns Nucleotide_acc, pfam, group and id. Expected counts ($\:{m}_{i,l}^{{\prime\:}}$) were determined by multiplying total Pfams x $\:{f}_{l}$ ($\:{m}_{i,l}^{{\prime\:}}=total\:\:{f}_{l}$), where ($\:{f}_{l}$) is the fraction relative to the total number of Pfams ($\:{f}_{l}=\frac{{n}_{l}}{N}$). $\:{s}_{i}^{{\prime\:}},l$ is the standard deviation (SD) for expected counts of the test group ($\:{s}_{i}^{{\prime\:}},l=\:\sqrt{tota{l}_{i}\:{f}_{l}\:(1-{f}_{l})}$). The difference in observed counts vs. expected counts was used to determine over-represented Pfams. This was also done for the control dataset. Z-scores were calculated as follows: ($\:{Z}_{i,l}=\frac{ob{s}_{i,l}-\:{m}_{i,l}^{{\prime\:}}}{{s}_{i}^{{\prime\:}},l}$). This Z-score represents the number of standard deviations the count is from the mean. The log1p was calculated for each non-negative Z-score to handle both skewed datasets in terms of Pfam counts, reducing the impact of highly occurring Pfam counts within both groups. A high positive log-transformed Z-score is indicative of a higher-than-expected count. A log-transformed Z-score was used for its suitability at looking at over-representation within a non-normally distributed dataset and plotted using ggplot2 v3.5.1 using R v4.4.2.

### Gene ontology, DRAM and mobile-OG database

Gene ontology (GO) was used to decipher potential systems that protein families are a part of GO terms were mapped to Pfams using the pfam2go command from the ragp R package v0.3.5.9000 [72]. There are limitations to the use of GO terms to infer biological systems due to their simplicity, hence it was only used to guide further analysis rather than being a descriptive end point [27]. Mobile-OG annotations were mapped using “hmmsearch” from HMMER v3.3.1 with an E-value of 1e-30 [26, 65]. DRAM annotations were cross-referenced with metadata file from the DRAM database v2 [25].

### Plasmid prediction and genomic Island prediction

Assemblies for each genome containing the lanthipeptide BGCs were downloaded from National Center for Biotechnology Information (NCBI). Plasmids were predicted using Platon v1.7 and the setting “--mode accuracy” [73]. This tool was chosen based on its suitability to predict plasmid contigs in short read draft assemblies without relying on taxon-specific plasmid databases. GIs were predicted using IslandPath v1.0.6 [74]. We then cross-referenced plasmid contigs and GI start-end locations with BGCs. BGCs were then found to be on a predicted plasmid contig or within a GI boundary.

### Prediction of defence-systems, anti-defence proteins and toxins

DefenseFinder version v2.0.0 with the option “--antidefensefinder” to predict both defence systems and anti-defence systems for each of the 1412 bacterial contigs and control genomes [75]. The taxonomy was appended using the R package taxonomizR v0.10.5 which pulls taxonomic data from NCBI (https://cran.r-project.org/web/packages/taxonomizr/index.html). Heatmaps were plotted using the R packager ‘pheatmap’ v1.0.12. The overview of distance from a LanM encoding gene was plotted using “geom_density” using R v4.3 using the midpoint of LanM encoding genes and defence genes as predicted by DefenseFinder. Proteins encoding Acetyltransferase Pfams were blasted vs. the toxinome database using BLASTp v2.11.0 + with the parameters ‘-evalue 1e-05 -outfmt 6’.

### Pfam correlation with increasing sampling size

To examine the monotonic relationship between sample size (number of accessions sampled) and Pfam count (the frequency of specific Pfam domains) we subsampled 100 random BGCs and their surrounding 40 kb window. To assess the correlation between the number of Pfam domains and BGC samples, we applied a Spearman correlation. Scatterplots were created using ggplot2, and the Spearman coefficient was calculated using the “stat_cor” function with the “spearman” method in R v4.4.2.

### Randomised defence system likelihood calculation

A random 30-consective genes were extracted from 200 bacterial contigs and cross-referenced for the presence or absence of phage-defence systems as predicted by DefenseFinder version v2.0.0 [75]. Thirty genes were chosen as this represents the approximate size of DNA surrounding lanthipeptide BGCs in the test dataset.

### Visualisation of operons

BGCs and regions upstream and downstream were plotted using gggenomes v1.0.0 using R v4.4.2 [76]. Genes were annotated using their description from NCBI.

## Supplementary Information

Supplementary Material 1.

Supplementary Material 2.

## Data Availability

All accessions used in this study are available in the Supplementary Tables and Walker et al. [23] (10.1186/s12864-020-06785-7) and are available from NCBI RefSeq. Scripts used to perform this analysis are stored in the GitHub repo “https://github.com/DEHourigan/co_localisation_lanthipeptide”. Accessions that are negative for lantibiotic genes and not found in Walker et al. [23] are found in Supplementary Table S13.
