# Supplementary material for: Colocalisation of lanthipeptide production with genetic exchange and defence systems across prokaryote genomes
Source: BMC Genomics. 2025 Dec 18;26:1108. doi: 10.1186/s12864-025-12219-z (PMC12715929; doi:10.1186/s12864-025-12219-z)
Supplement: Supplementary file 1 — Supplementary Material 1. [file 12864_2025_12219_MOESM1_ESM.docx]

# Supplementary text file.

David Hourigan^a,b^, Colin Hill^a,b^, R. Paul Ross^a,b,c^#

^a^ APC Microbiome Ireland, Biosciences Institute, Biosciences Research Institute, College Rd, University College, Cork, Ireland

^b^ School of Microbiology, University College Cork, College Rd, University College, Cork, Ireland

^c^ Teagasc Food Research Centre, Moorepark, Moorepark West, Fermoy, Co. Cork, Ireland

#Address Correspondence: [p.ross@ucc.ie](mailto:p.ross@ucc.ie)

**
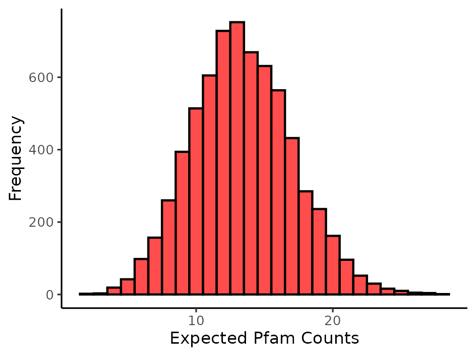
**

**Figure S1. Random distribution of Pfam counts given the size of the starting dataset.** Pfams from the lanthipeptide regions were made non-redundant (n=6767). Random counts were sampled mirroring the same size dataset as the dataset.


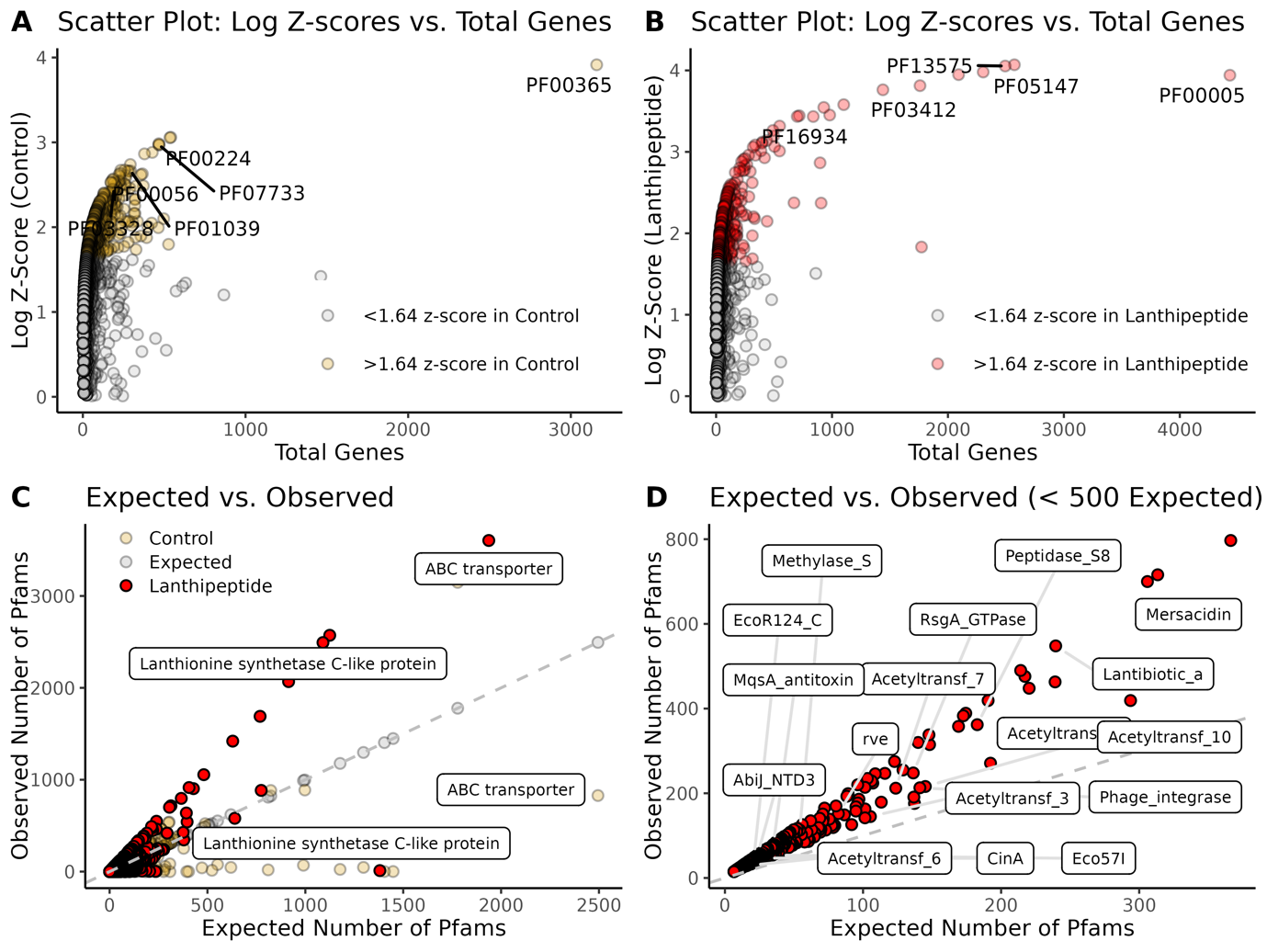


**Fig. S2** **Over-represented Pfams near lanthipeptide BGCs compared to Pks. (a)** A scatter plot of log(1 + x)-transformed Z-score counts of Pfams in the control dataset (e.g., PF00365). Highlighted in gold are Pfams that have a Z-score >1.64. **(b)** A scatter plot of log(1+x)-transformed Z-score counts of Pfams in the test dataset (PF05147). Highlighted in red are Pfams that have a score >1.64. These Pfams include ABC transporter (PF00005), Domain of unknown function (DUF4135/LanC-like; PF13575), Peptidase C39 family (PF03412) and the lanthipeptide Mersacidin (PF16934). These Pfams are commonly found in class II lanthipeptide BGCs. **(c)** Plot of expected Pfam counts for the test group, a control group and expected data (grey). In this plot the grey line represents simulated expected data give the size of the dataset i.e. an increase in observations with an increase in expected observations. Everything above this line has higher counts in the observed dataset than expected. Red circles represent Pfam counts within the lanthipeptide group. Gold circles are counts of the same Pfams in the control dataset. Grey circles represent expected counts for expected data. The Pfam Lanthionine synthetase C-like protein has counts for both Control and Lanthipeptide groups, where counts for Control are below the expected value and the counts for the test group are above the expected value. **(d)** This plot is a subset of plot (b) where only positively correlated and statistically significant Pfams near lanthipeptide BGCs are shown between the ranges of 800 observed counts and 300 expected counts. Multiple protein families associated with defence against exogenous DNA were observed in the dataset. Some of which are shown. The distribution of protein family occurrence between test and control datasets was investigated. In the control dataset (Fig. 3a), several Pfams exhibit a log transformed Z-score greater than 2, including PF07733 (Bacterial DNA polymerase III alpha NTPase domain) and PF00224 (Pyruvate kinase, barrel domain), alongside PF00365 (PFK). In the test dataset (Fig. 3b), Pfams such as PF00005 (ABC transporter), PF13575 (Domain of unknown function, DUF4135, encoding a LanC-like protein), and PF03412 (Peptidase C39 family) are notably over-represented, with PF05147 (LanM) also observed. In total, 189 Pfams with a log-transformed Z-score greater than 2 were identified (Table S1). A plot of observed *versus* expected counts was created (Fig. 3b). The observed *versus* expected data for both the control and test datasets were plotted and two distinct Pfams associated with LanM-type bacteriocin production are highlighted (Fig. 3c). An expected observation is the main over-represented Pfams are ABC-transporters (PF00005) and the LanM domain (lanthionine synthetase C-like). A dataset where expected and observed counts are in alignment is signified in grey.

**(b)**
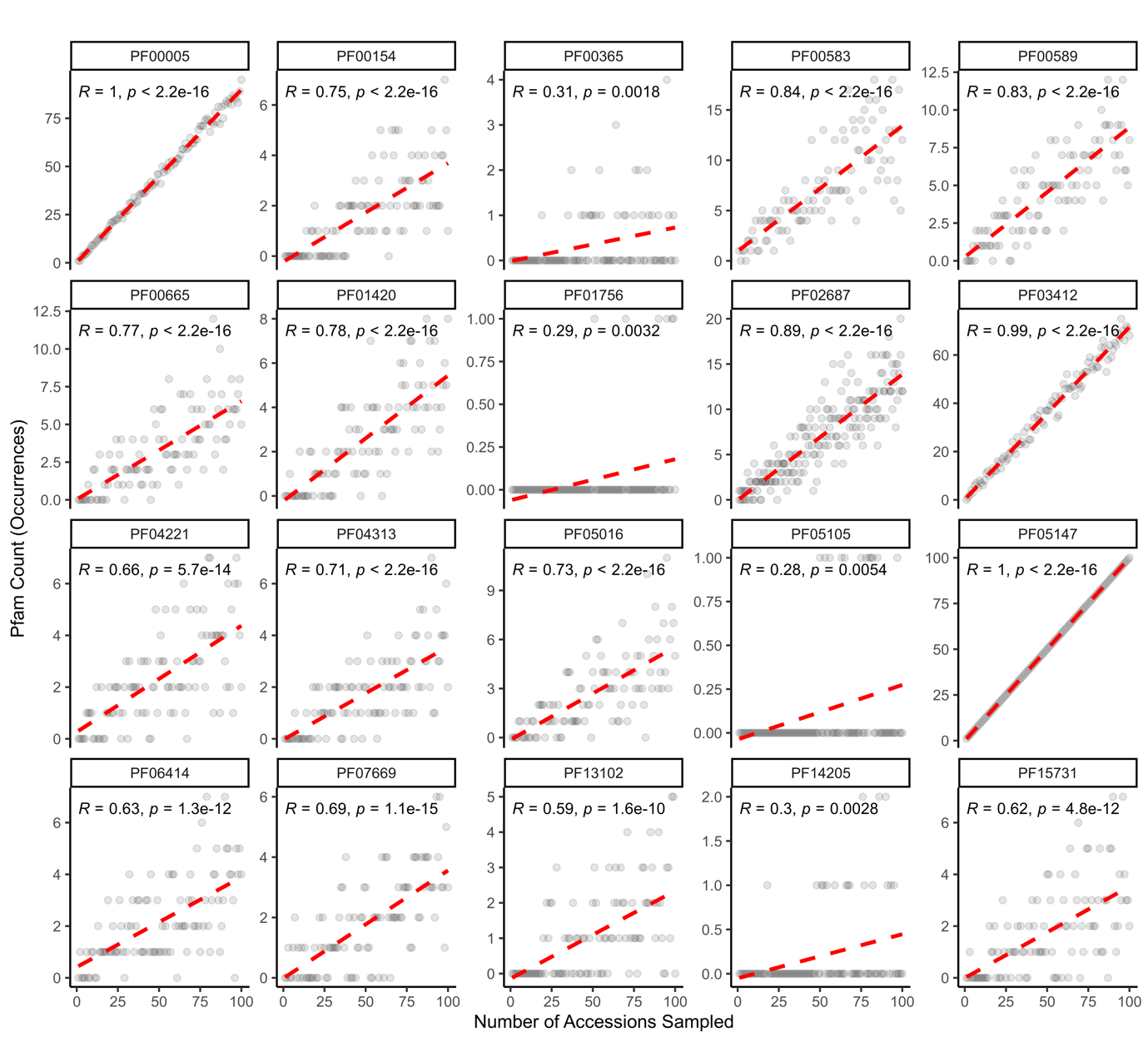
 **Figure S3. (a) Accumulation curve for Pfams deemed of interest (b) Spearman correlation between region counts and Pfams deemed of interest.** Pfam domains that were selected as negative controls: PF00365 (Pfk) and randomly selected PF01756, PF05105 and PF14205. Transport-related domains included PF02687 and PF00005. PF03412 represents a peptidase domain, and PF05147 corresponds to LanM, an enzyme involved in lanthipeptide biosynthesis. Toxin-antitoxin system components included PF05016 (ParDE toxin), PF06414 (Zeta toxin), PF04221 and PF15731 (antitoxins), and PF00583 (acetyltransferase, sometimes implicated in resistance mechanisms). Restriction-modification system-associated domains were PF01420, PF07669 (Eco57I methylase), and PF04313 (HsdR_N, Type I restriction enzyme subunit). Mobility-associated elements included PF00589 and PF13102 (phage integrases), PF00665 (recombinase), and PF00154 (RecA, a key recombination protein).

**
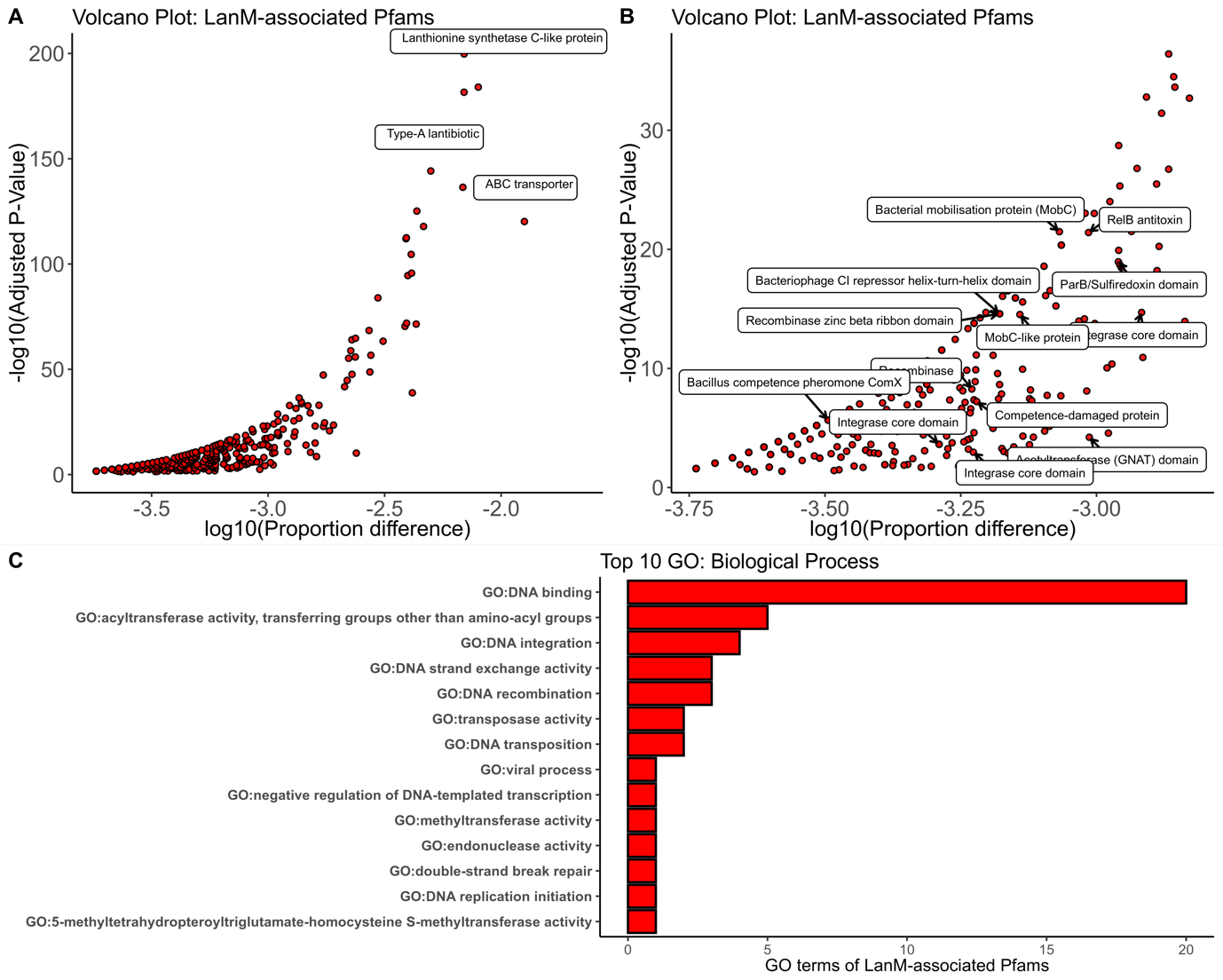
**

**Fig. S4 Statistically over-represented Pfams are associated with the movement of DNA and protection against DNA. (a)** Statistically over-represented Pfams that co-occur with LanM in comparison to PFK **(b)** Statistically over-represented Pfams that co-occur with LanM in comparison to PFK below a -log10(adjusted P-Value) of 40. **(c)** Top 14 Gene ontology (GO) terms associated with movement of DNA are shown. GO terms were mapped to each statistically over-represented Pfam and the counts of Pfams that fall within a GO-term are shown. There are GO processes mapped to Pfams near lanthipeptide BGCs that include peptide modification, bacteria defence systems, DNA-templated transcription, phosphoenolpyruvate-dependent sugar phosphotransferase system (not shown), DNA transposition and DNA modification (shown).

**
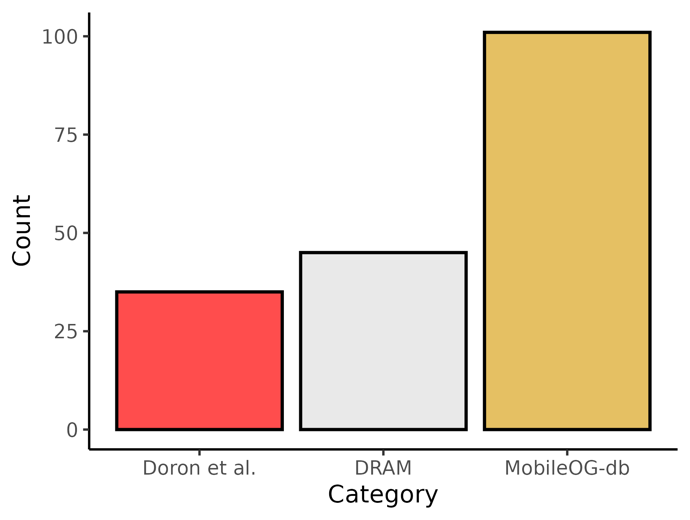
**

**Figure S5. The number of enriched Pfams found in Doron et al (2018) and the DRAM and mobileOG databases.**

**
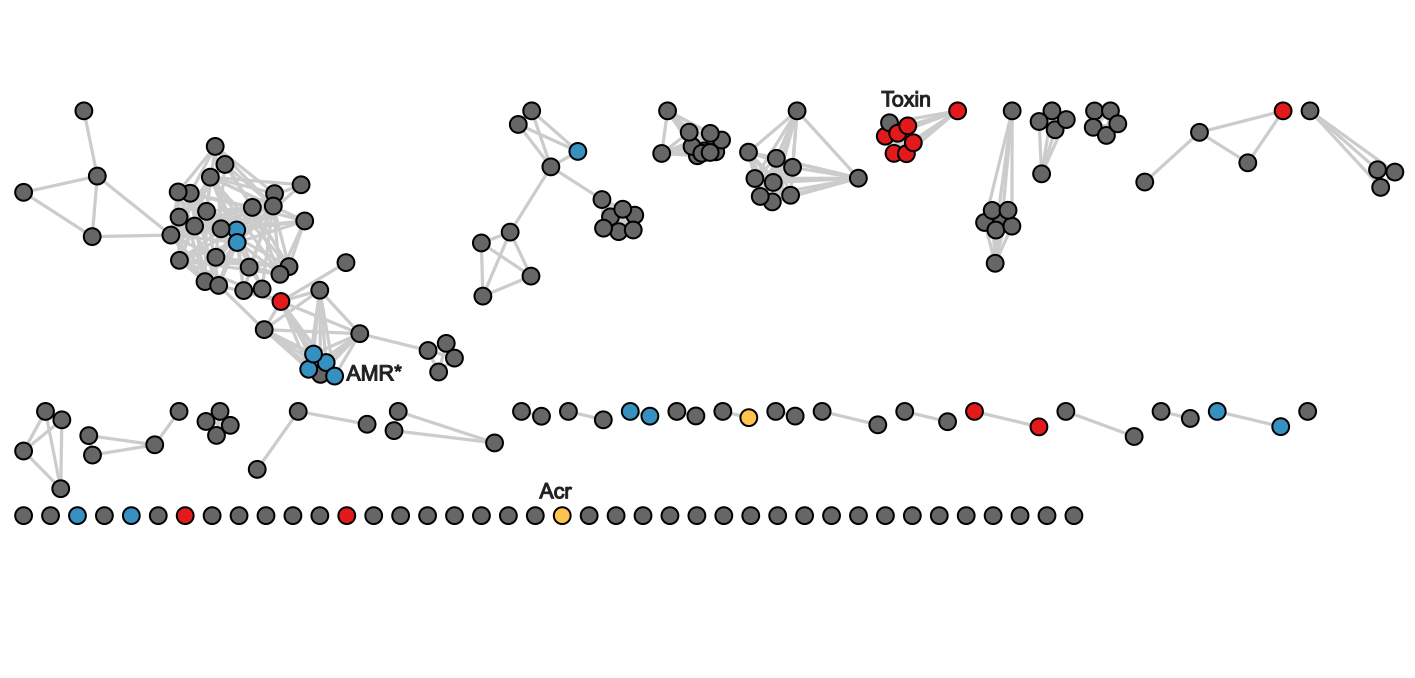
**

**Figure S6. The number of enriched Pfams found in Doron et al (2018) and the DRAM and mobileOG databases.** Shown is a sequence similarity network for proteins containing GNAT acetyltransferase domains. These are coloured based on their putative function such as Toxin (red), AMR gene (blue)* and anti-CRISPR protein (yellow). *These do not share high %ID to characterised AMR proteins so their function is putative.


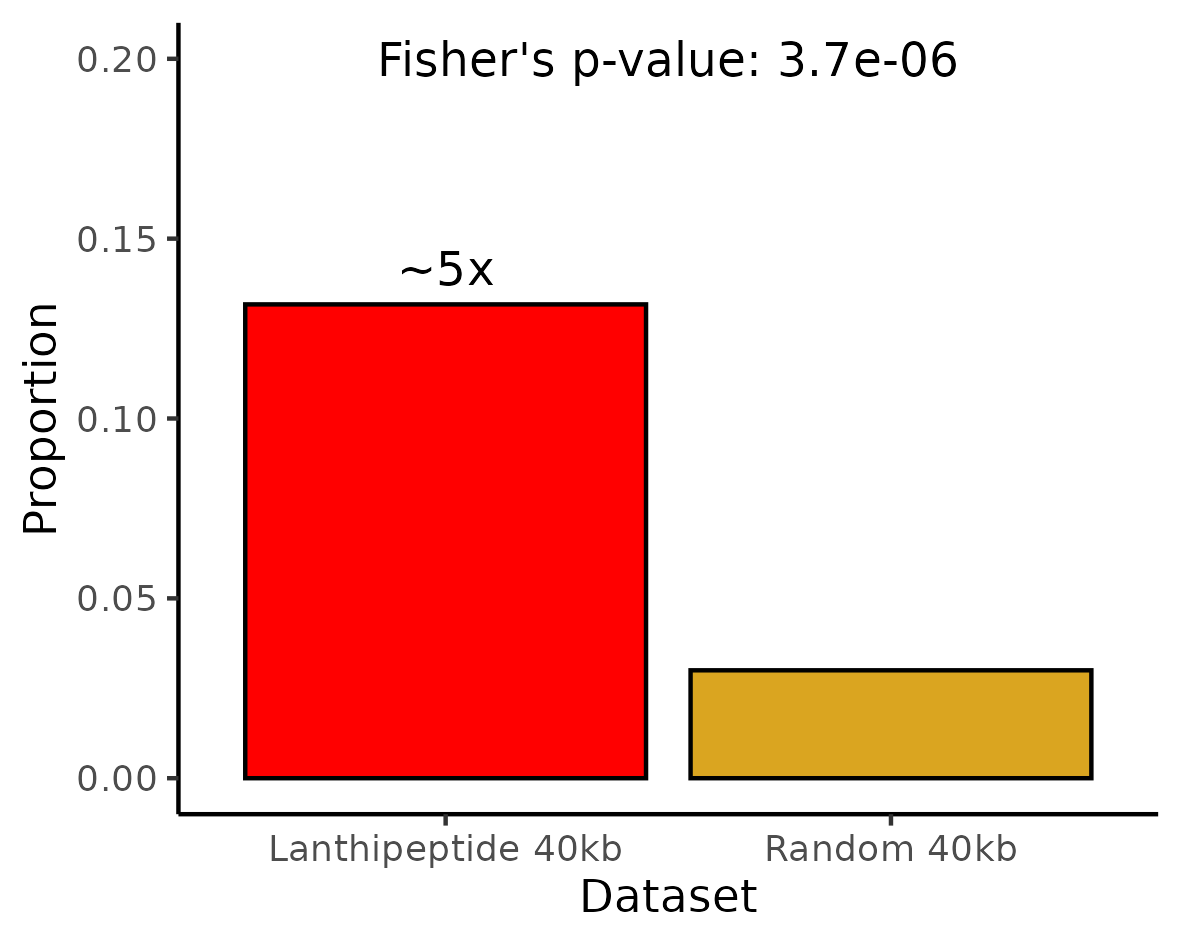


**Figure S7. Fold-likelihood of observing a phage defence system near a BGC than a random region of genomic DNA.**

To test whether anti-phage defence systems are more frequently located near lanthipeptide BGCs compared to random genomic regions with similar coding density, we analysed defence system occurrences in 40 kb windows surrounding lanthipeptide versus random genomic regions. In total 186 out of 1412 BGCs were localised near a defence system whereas the 6 out of 200 regions encoded a defence system (3.0%). Fisher’s Exact Test revealed a significant enrichment of defence systems near BGCs (p = 3.7 × 10⁻⁶), with an odds ratio of 4.90 (95% CI: 2.17–13.72). This indicates that defence systems are nearly five times more likely to be found near lanthipeptide BGCs than in comparable random regions.


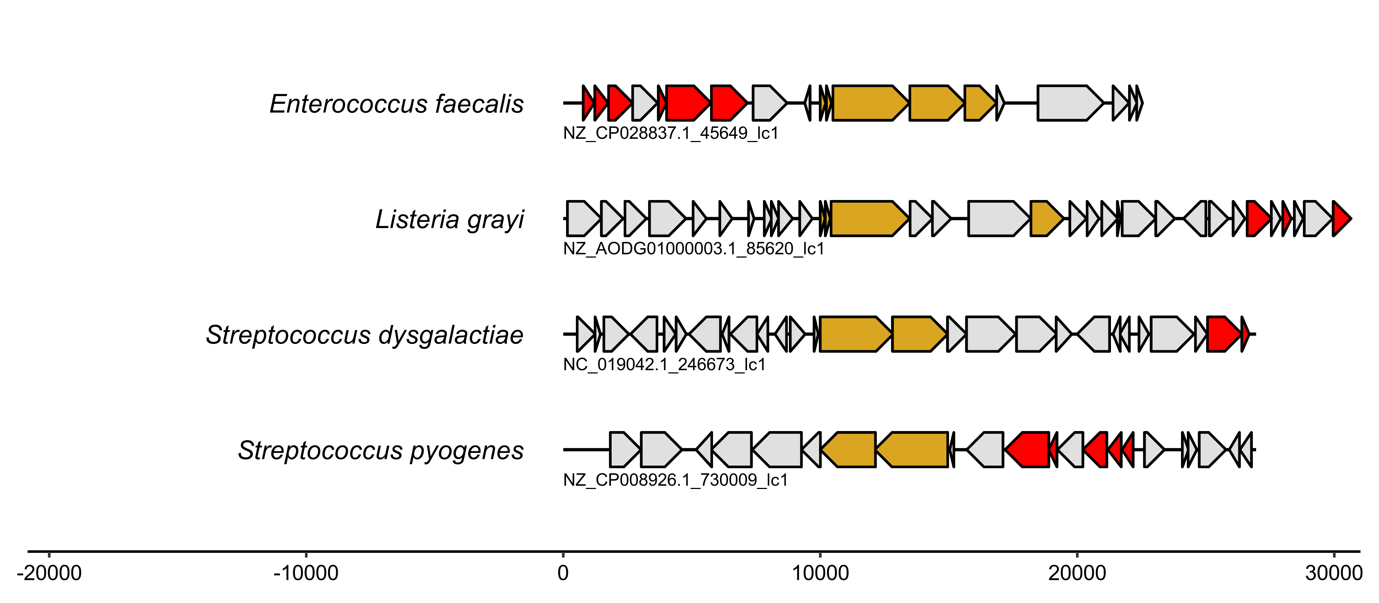


**Fig. S8. Sugar utilisation systems enriched near class II lanthipeptide BGCs.**

*E. faecalis* (NZ_CP028837.1) encodes the class II lanthipeptide cytolysin and is located less than 3kb from a PTS transport system predicted to transport galactose/tagatose. *S. dysgalactiae* (NC_019042.1) encodes a class II lanthipeptide BGC upstream from a PTS sugar transporter. *S. pyogenes* (NZ_CP008926.1) encodes a BGC co-localised with both a PTS system and an RM-system. *Listeria gray* (NZ_AODG01000003.1) encodes a BGC localised next to a fructose PTS system.


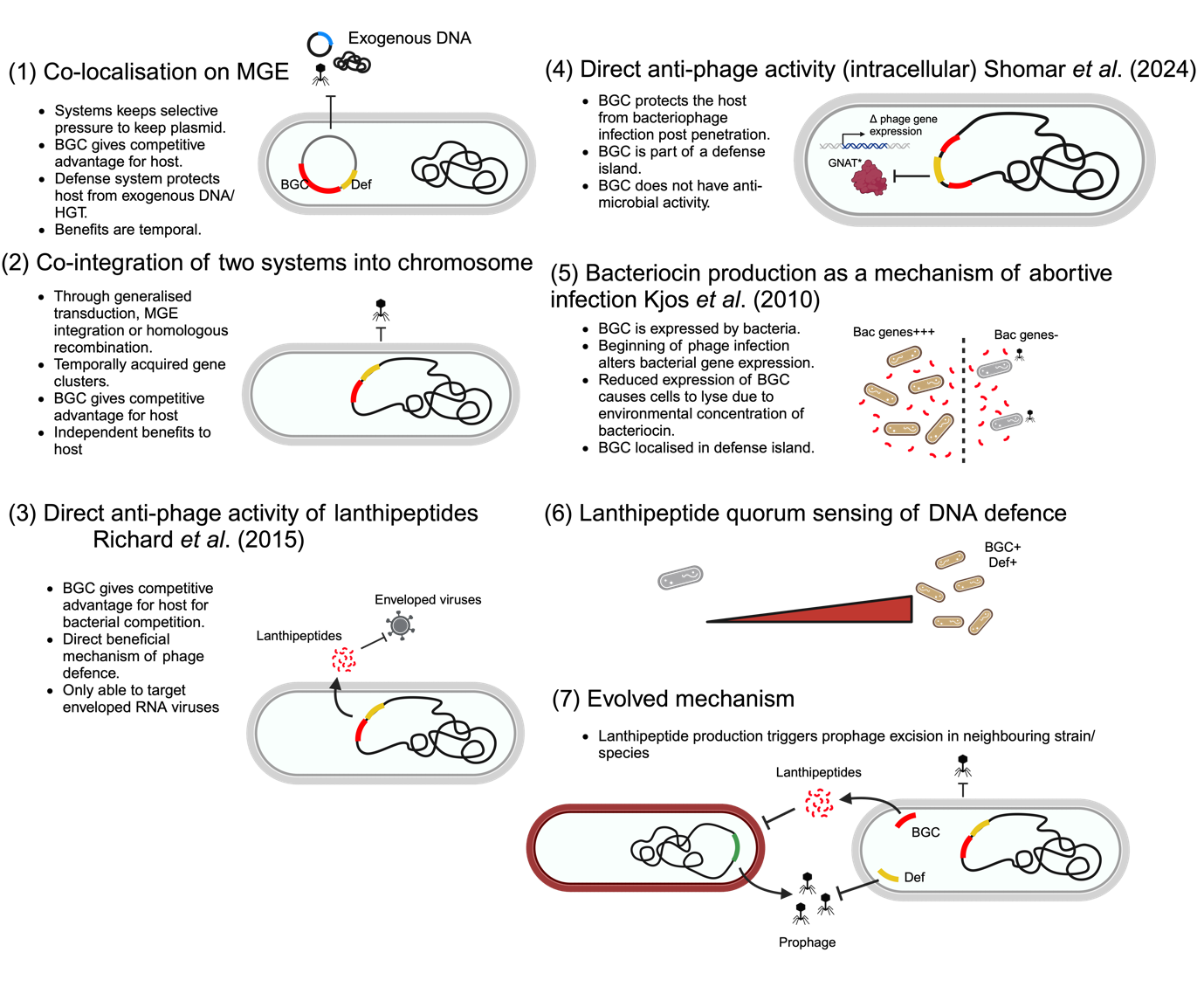


**Fig. S9. Proposed benefits to the host that contains co-localised lanthipeptide BGC and a DNA defence mechanism.**


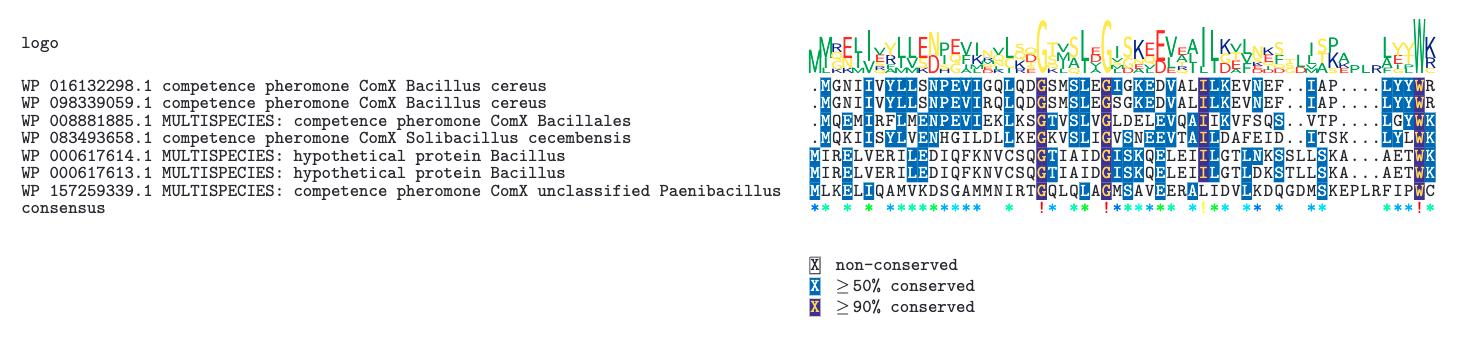


**Fig. S10. Unique ComX proteins from Bacilli.** Competence pheromone peptides were identified in *Paenibacillus*, *B. cereus* and *Solibacillus cecembensis*. All predicted peptides have a C-terminal tryptophan that is known to be highly conserved among ComX proteins.
